# Jon Sarkin: the eternal now

**DOI:** 10.1017/S2045796023000227

**Published:** 2023-04-24

**Authors:** Colin Rhodes

**Affiliations:** College of Fine Arts, Hunan Normal University, Hunan, China

**Figure 1.**
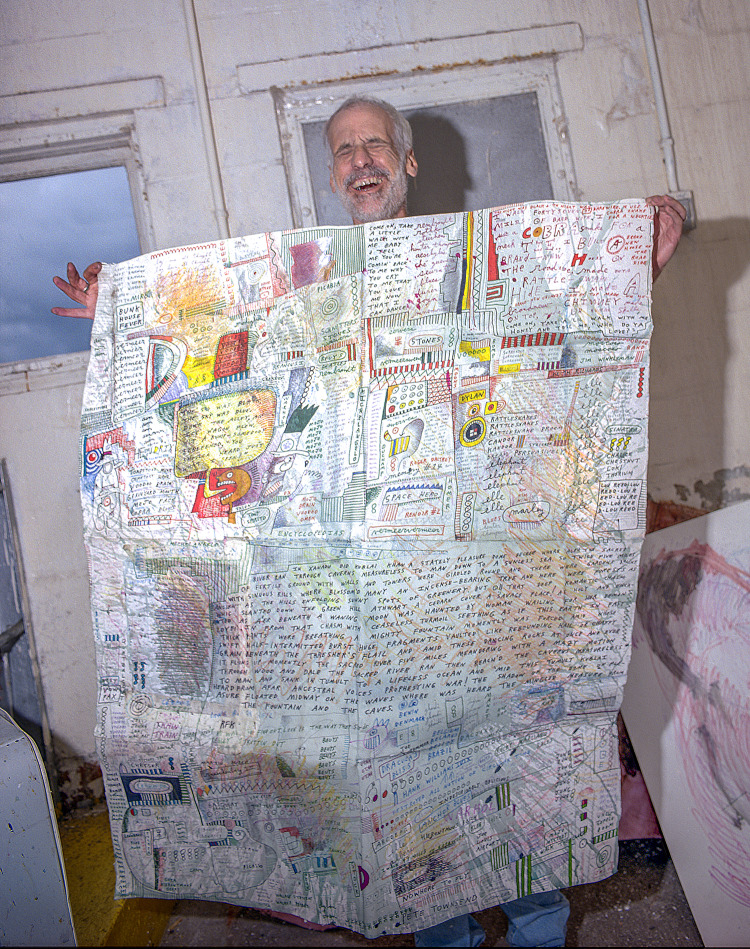
Jon Sarkin in his dockside studio, Gloucester, MA, 2021. Photograph by Ted Degener

Jon Sarkin (b.1953) (Figure 1) is a remarkable contemporary American artist whose work stands comparison with some of the most prominent names of the last six decades, particularly in that vein which critically embraces pop culture, from Jasper Johns and Robert Rauschenberg to Jean-Michel Basquiat and Cy Twombly. Self-taught as an artist, he draws his creative imagery from the depths of his unconscious and uses the kinds of automatic methods beloved of Surrealism, Neo-Dada and the Beat generation writers. Working compulsively and obsessively, Sarkin has produced a large body of visual art and textual work that is both profound in its accumulated cultural messaging and truly arresting visually.

**Figure 2.**
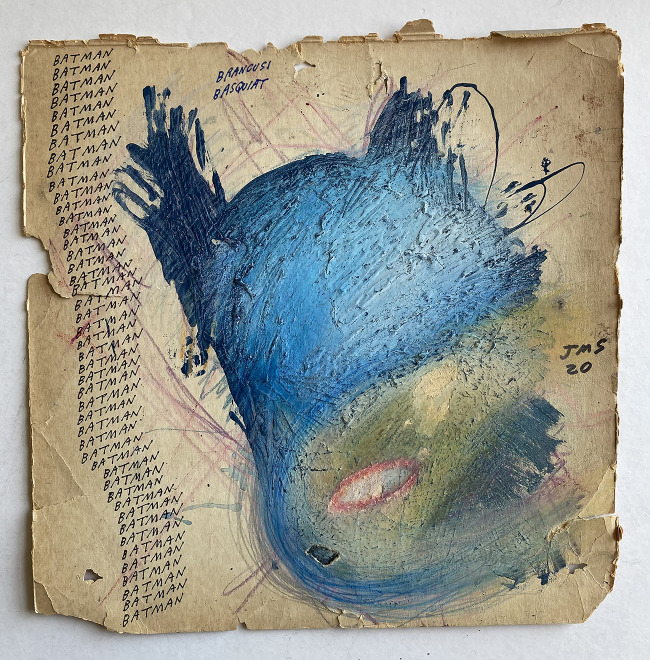
Jon Sarkin, *Batman*, 2020, mixed media on found cardboard, 31 x 31 cm. Image courtesy the Henry Boxer Gallery, London

Each individual artwork is part of a continuum beginning more than three decades ago, and which will only halt when Sarkin is no longer able to engage in the daily act of making. The work as a corpus is dense in its signification and critical revelations; it is highly literate and emblematic of the Pop culture that defined America – and in some sense most of the rest of the world – in the second half of the last century. Some motifs recur constantly, from Batman (of the mid-1960s TV series, rather than the dark, gothic figure of his comic-book roots and later movies) (Figure 2) and other figures that owe their roots to drawings by Robert Crumb and other underground comics that Sarkin devoured in his younger days to seemingly endless variations of the potted cacti and aloe vera plants redolent of mid-century middle-class domestication, similarly to the litany of names that appear across his work, reflecting the artist’s artistic, literary and musical interests. Yet, this is no chronology, unfolding part by part; it is a mess of stuff. Sarkin embraces and positively utilizes fragmentation as process and way of life. As he says, ‘I think about many things. My thoughts are all jumbled. This is a good thing in terms of my art. In terms of everyday life? hmm…. I carry this jumble with me. Over and over I think about stuff, reminiscing, planning, stuck in the eternal now’ Sarkin (2022d). The accumulation of word and image across 30 years of art practice thus amounts to a kind of history in synchronic time.

Sarkin’s oeuvre has been generated and achieved in the wake of a kind of creative and psychological epiphany grounded in a catastrophic medical episode that occurred in his mid-thirties, and which saw his life transformed emphatically and permanently. Until that moment, Sarkin’s life journey had been marked only by its middle-class conventionality: a successful chiropractic practice, home, family, golf and occasionally jamming with his guitar in a local bar. Then, in October 1988, he experienced a strange shudder in his head while playing golf, which developed subsequently into unbearable tinnitus and hyperacusis. The cause was eventually identified as a swollen blood vessel impinging on the cochlear nerve in the left side of his brain. In August the following year, he underwent an operation to correct this. Initially, it seemed to have been successful, but hours later Sarkin suffered a massive stroke, which resulted in much of the left side of his brain being destroyed. He remained in a critical state for months, hovering between life and death, and when he did begin to emerge from the trauma, it was clear that much had changed for him – physically and psychologically – forever. He had become deaf in his left ear and experienced constant double vision. The most thorough narrative and psychological analysis of Sarkin’s traumatic injury and his subsequent recovery journey is Nutt (2011). The most palpable change in the current context was the emergence of a new and obsessive urge to make art, which quickly became a prime driving force.

Art-making and writing were the ways in which Sarkin tried to make sense of and fix the overwhelming cacophony of sensations and memory that assailed him now. ‘I looked at one of my older works’, he told me, ‘and it said, “I require a sphere of infinite volume in which to house my ideas”’ Sarkin (2023). He attributes the thought to a snatch of Bob Dylan’s *From a Buick Six*: ‘I need a dump truck, mama, to/Unload my head’. Images and text emerge, as it were, more or less directly from a visceral psychological level, often fragmentary and iridescent. ‘My brain [now] works … in a decidedly clunky fashion’, he notes, ‘My stroke and my art are so, SO conjoined’ Sarkin (2022f). Sarkin describes his practice as ‘stream-of-consciousness’, which is in line with his tendency to having no filter in his everyday conduct. He recognizes that his ‘art and writing start somewhere atavistically and end up more so’ Sarkin (2022a).

Each new work begins with a mark, which may be either a word or image, and develops automatically from there. Pieces are worked on concurrently and earlier works often returned to, and new layers added – in his paintings, this often results each time in the complete obliteration of earlier images, as with a mural, titled *Unbound*, that he created for the Mullen Lowe advertising agency, Boston in 2009, and whose genesis and progress can be seen in a film made at the time McGrath and Pearson (2011). There is a very real sense that a work is only ‘finished’ when the artist no longer has it in his possession. Sarkin’s art, then, is simultaneously an existential and aesthetic endeavour; it is an act of making sense of the world and regaining equilibrium and also a pursuit of the eradication of dualities through creative revelation. As he puts it, ‘I attempt to heal my unhealable wound knowing that I can never accomplish this, yet I persist’ Sarkin (2022d). Moreover, he is clear that ‘art has become my life and my life is my art’ Sarkin (2022c). He maintains a strict work schedule, centred around his Gloucester studio, to which he travels daily. Yet his emergence as an artist has not always been easy, coming as he does, from a social context that does not really accept the notion of the profession of ‘artist’; always there is a lingering suggestion that he must surely have lost, rather than gained from no longer having a ‘regular job’.

**Figure 3.**
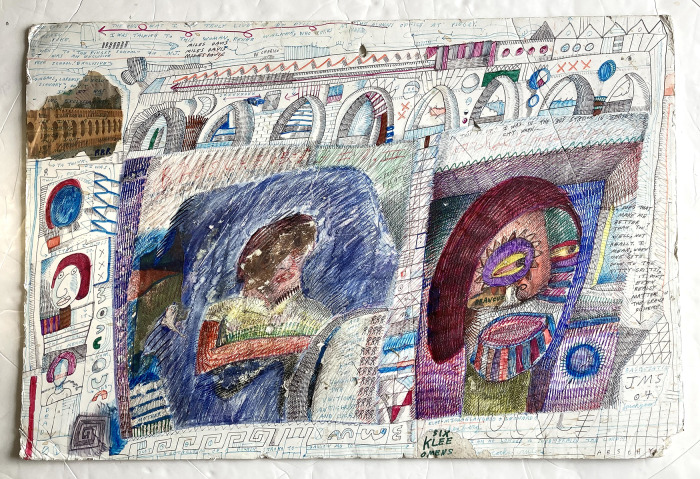
Jon Sarkin, *Six Klee Omens*, (2004), mixed media on foamboard, 71 x 51 cm. Image courtesy the Henry Boxer Gallery, London

For some time, Sarkin’s favoured surfaces have been artist’s foamboard and the backs of 12-inch album sleeves. Foamboard is more usually employed for mounting drawings on paper, or backing for framing, but its durability lends itself perfectly to the physicality of his method. A work like Six Klee Omens (2004) (Figure 3) clearly displays the evidence of scratching, carving, tearing and spilling that are as much a part of their pictorial development as the more conventional mark-making and writing they contain. Disassembled record covers are also a favourite base, as in the case of *Batman*, since the cardboard is reasonably strong, and the dimensions and square format seem congenial to the worlds of text and image he characteristically produces, including regular musical references, from the blues, rock and pop to classical and jazz. Sourced from his local record store, which gives away free any unplayable and unsellable vinyl, he can obtain them in the kinds of quantity that allows him to work in series. Interested only in the unprinted insides of the covers, Sarkin tosses the unwanted vinyl in piles in his studio, where it accumulates until someone is willing to throw it away.

At times, the textual content of works consists of more or less fluid narrative, as in *Six Klee Omens*, which relates some thoughts about his privileged past. The sense of a stream of consciousness unfolding is strong, with text meandering across the whole surface, punctuated by ‘illustrations’ and arrows that seemingly assist the reader with directions. But it is still easy to get lost, and the sheet is also scattered with other references; to artists, musicians and writers. It is clear that different elements of text were written at different times; a block of text above the large area of blue at left centre has been all but obliterated by later scraping and scribble, which reminds viewers of Sarkin’s habit of returning continually to works and enacting a process of layering that enriches and complicates the developing imagery and text, challenging teleology. It is even unclear in many cases whether the dates written on the front of works refer to when they were ‘completed’, or whether they belong to some earlier stage in the process.

**Figure 4.**
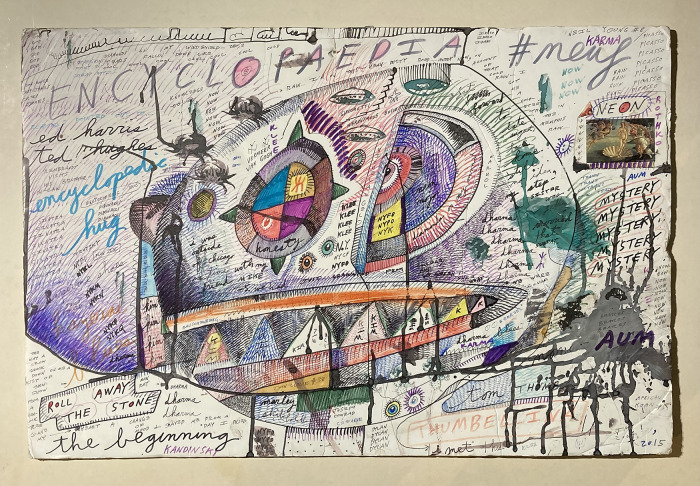
Jon Sarkin, *Roll Away the Stone*, (2015), mixed media on foamboard, 71 x 51 cm. Image courtesy the Henry Boxer Gallery, London

A dominant tendency is to put blocks of words together in list form, sometimes as a repeated single noun. In the record cover works, a single word is often repeated, mantra-like, as in Coltrane (2021), which consists entirely of the great jazz saxophonist’s name repeated 128 times around a set of visual forms that may be figurative or abstract, but which certainly suggest the musical forms of bebop in their realization. Words can also be subject to change as they multiply: for example, in Klee (2015), a list begins with the name of another jazz great, Charles Mingus, and is altered through improvization and changes in visual phrasing, before returning to the original: ‘MINGUS, MINGUS, MINGUS, GIMNSU, IUGMNS, MUNGIS, MINGUS, MINGUS, MINGUS’. Such word lists appear simultaneously, across different areas in the foamboard works. Roll Away the Stone (2015) (Figure 4), for example, is rich in allusion, including repetition lists of ‘Dylan’, ‘Dharma’, ‘Picasso’, ‘Sinatra’ and others. The word ‘Kim’ – the name of Sarkin’s wife – also appears five times, in a series of triangles that may be pyramids, boat sails or teeth; or more likely all of those things at once. In a sixth and seventh triangle, the name is reduced to ‘K’, and below that are the words ‘Dharma’ and ‘Karma’. Sarkin offers the following explanation of his method: ‘This is how my mind works: “Crete” rhymes with “Magritte”. Coincidence or synchronistic event? Hmm … probably both simultaneously. Insight: are you familiar with “Schroedinger’s cat” thought experiment? A cat is put in a box. There’s a button you can press. It’s either (1) a dummy switch where nothing happens; or (2) it releases a poisonous gas and kills the cat. Before one opens the box, the cat’s both alive and dead. Shades of Philip K. Dick. The Crete/Magritte shtick’s both random and meaningful at the same time’ Sarkin (2022b).

Precisely because his technique approaches something like the ‘pure psychic autonomism’ that was the holy grail of classic surrealist creativity, Sarkin is reticent about revealing his methodologies even to himself. ‘My art’s all about UNCERTAINTY … always feeling the inevitable neurotic twang that accompanies unknown-questing’ Sarkin (2022f). Viewers engage with a Sarkin artwork or series of works like wanderers through some uncharted landscape whose individual elements are familiar, but whose totality is without prescribed orientation. The artist tells us, ‘Now that it’s been over 34 years since the stroke, my perception of reality’s gradually “adapted” and now the main insight I have regarding my transformation is … the perception of mutually exclusive polar opposites as comfortably co-existing’ Sarkin (2022e). In fact, there is no ‘path’ or linear narrative to be discovered in these works; they contain multitudes, to paraphrase Walt Whitman and, more recently, Dylan, but synchrony is hardwired in them.
